# Co-morbidities associated with molar-incisor hypomineralisation in 8 to 16 year old pupils in Ile-Ife, Nigeria

**DOI:** 10.1186/s12903-015-0017-7

**Published:** 2015-03-13

**Authors:** Titus Ayodeji Oyedele, Morenike O Folayan, Comfort A Adekoya-Sofowora, Elizabeth O Oziegbe

**Affiliations:** Department of Child Dental Health, Obafemi Awolowo University Teaching Hospitals Complex, Ile-Ife, Nigeria; Department of Child Dental Health, Obafemi Awolowo University, Ile-Ife, Nigeria

**Keywords:** Hypomineralisation, Co-morbidities, Dentine sensitivity

## Abstract

**Background:**

This study aimed to identify the prevalence of oral co-morbidities in 8 to 16 years old children with Molar Incisor Hypomineralisation (MIH) and compare this with the prevalence of same oral lesions in children without MIH.

**Method:**

Study participants were selected through a multi-staged sampling technique. The children were asked if they had dentine hypersensitivity or any concerns about their aesthetics. Children were examined for MIH, caries, traumatic dental injury and their oral hygiene status. The association between MIH and each of the independent variables was determined.

**Results:**

Only children with MIH had aesthetic related concerns and dentine hypersensitivity. The differences in the oral hygiene status (p < 0.001) and caries prevalence (p < 0.001) of children with and without MIH were statistically significant. The prevalence of traumatic dental injury did not differ statistically between children with MIH and those without MIH (p = 0.24).

**Conclusion:**

Children with MIH had more oral pathologies than children without MIH. These co-morbidities (dentine hypersensitivity, aesthetic concerns, caries and oral hygiene) are capable of impacting negatively on the quality of life of the children. Screening for children with MIH may help facilitate prompt access to treatment.

## Background

Molar-Incisor Hypomineralisation (MIH) is a lesion of systemic origin affecting one to four permanent first molars, and may affect the permanent incisors [1]. The risk of involving the permanent incisors increases as the number of permanent first molars affected increases [2].

Clinically, the defect caused by MIH appears as white, yellow or brown discolouration, often affects the occlusal surfaces of the first permanent molars and may be more frequent in the maxilla than mandible [3-5]. The defective enamel can cause tooth sensitivity, disfigurement, rapid plaque retention, caries and its sequelea [6,7]. This causes considerable discomfort for the child, it can be of great concern to the parents, and pose management problems for the clinician [6].

Unfortunately, the affected molars are more difficult to anaesthetise due to the subclinical inflammation of the pulpal cells, resulting from porosity of the enamel, which allows bacterial toxins to penetrate and cause pulpal reactions [6]. Due to the difficulties in achieving adequate anaesthesia and the frequent treatments required, children with MIH also run the risk of developing dental fear and anxiety [6]. Continuous disintegration of the enamel of the affected teeth, and difficulties with bonding with dental materials [8,9], suggests that children with MIH require extensive and often repeated restorative treatment, especially on the molars [6].

There are still very few studies on MIH, especially in Africa. The aim of this study is to determine the prevalence of MIH and its co-morbidities in children in Nigeria. Specifically, the study will determine and compare the proportion of children with or without MIH who has concerns with tooth sensitivity, aesthetic concerns, caries, poor oral hygiene and traumatic dental injury. The findings should help improve clinicians’ diagnosis, and prevention therapies instituted in children with MIH.

## Methods

### Study population

This study was across-sectional study which recruited children aged eight to sixteen years, resident in Ife Central Local Government of Ile-Ife, a sub-urban town in the south-western Nigeria. Pupils whose legal guardian consented to their participation, and those who gave assent to study participation, were eligible to participate in the study. Only those who had fully erupted permanent first molars and incisors were enrolled. Children with hypodontia, anodontia and amelogenesis imperfecta were excluded from the study.

Study participants were selected through a multi-staged sampling technique that resulted in the selection of a representative sample of children from all the socioeconomic strata. Selection of study participants through a proportional representation of public and private schools in the sampling frame helped to ensure that children from all the socioeconomic strata were recruited for the study.

### Sampling technique

First, Ife Central Local Government was divided into three geographical areas each consisting of four political wards. One political ward was selected from each of the geographical areas by ballot. Second, in each ward, the schools were stratified into public primary, private primary, public junior secondary, private junior secondary, public senior secondary and private senior secondary schools respectively. A list of schools obtained from the Osun State Ministry of Education was used for the stratification. One school was randomly selected from each strata by balloting. In effect, six schools were randomly selected from each ward and 18 schools from Ife Central Local Government were used for the study. Third, the lists of the children in each class in each of the selected schools were reviewed. Classes with the high numbers of children who met the age criteria for the study were selected for study participation. All selected study participants were invited to participate in the study and were given informed consent forms for their parents.

### Sample size

The sample size was determined by the statistical formula proposed by Araoye [10]. The estimated proportion of children with MIH was 40%, using the highest reported prevalence from various studies reported [5]. The minimum sample size for the study population was 405 children. To ensure adequate representation of children and to allow for meaningful subgroup analysis, all children who met the inclusion criteria at the recruitment sites were recruited for the study.

### Data collection

The data collection tool captured details of the age and sex of each child. Respondents were also asked if they had tooth sensitivity and if they were satisfied with their tooth appearance. Specifically, children were asked whether they could feel any shocking sensation while drinking water, any cold drink and or while eating, to determine presence of absence of dentine hypersensitivity. Each child was also asked about their satisfaction (or lack of it) with the appearance of their teeth. Respondents who showed concern were noted as having concerns about their aesthetics, while those who showed no concern were noted as having no aesthetic concerns.

### Intra-oral examination

Intra-oral examination was conducted for each respondent under natural light while seated on the classroom chair. The teeth were examined wet after debris was removed with gauze. Each tooth was examined for MIH, caries, and traumatic dental injury. The status of oral hygiene was also assessed.

### Molar-incisor-hypomineralisation

A diagnosis of MIH was made based on the criteria described by Jalevik [5]. First, the presence of absence of MIH was determined for each child. For children with MIH, affected tooth were identified. Foe each affected tooth, the severity (mild, moderate or severe) of the lesion was also identified. Mild lesions were those with demarcated opacities present in the non-stress bearing areas of the molars with no enamel loss from fracturing [5]. Moderate lesions were those with presence of any atypical restoration, demarcated opacities present on the occlusal/incisal third of the teeth with no post eruptive enamel break down or with post-eruptive enamel breakdown limited to one or two surfaces without cuspal involvement [5]. Severe lesions were those with post eruptive enamel breakdown [5]. The severity of the lesion for each child was defined by the most severe defect on the affected tooth/teeth. All children diagnosed with MIH would have at least one molar was affected, with or without the involvement of the incisors.

### Caries

Caries was diagnosis using the World Health Oral Health Survey recommendations [11]. Each tooth was examined for dental caries using a plane mouth mirror, using natural light while the child was seated on a chair. Caries status was assessed using the Decayed Missing and Filled (DMFT) index. Decayed (D) teeth were defined as any tooth whose crown had an unmistakable cavitation on the pits or fissures, or on a tooth surface or a filled crown with decay, when it has one or more permanent restorations that are decayed. The F was defined as a filled crown with no decay, when it has one or more permanent restorations, and there is no caries anywhere on the crown. The M was defined as a missing tooth due to caries; when a tooth has been extracted due to caries. To arrive at a DMFT score for an individual patient’s mouth, three values must be determined: the number of teeth with carious lesions, the number of extracted teeth due to caries, and the number of teeth with fillings or crowns [12]. The number of teeth are then summed together to give the DMFT score for the permanent dentition.

### Traumatic dental injury

Trauma to the anterior teeth of each study participant was determined using Ellis and Davey classification [13]. Trauma was classified as present when there was a simple fracture of the crown involving little or no dentine; extensive fracture of the crown involving considerable dentine and exposure of dental pulp, or loss of the entire crown.

### Oral hygiene status

Oral hygiene was recorded using the simplified**-**oral hygiene index (OHI-S) described by Greene and Vermillion [14]. The OHI-S has two components; the debris index and the calculus index. Each of these indexes in turn, is based on numerical determinations representing the amount of debris or calculus found on the preselected tooth surfaces. The calculus index simplified (CI-S) and debris index simplified (DI-S) value may range from 0 to 3, the OHI-S value which is the sum of CI-S and DI-S range from 0 to 6. Oral hygiene index score of 0–1.2 means good oral hygiene; 1.3-3.0 means fair oral hygiene and 3.0-6.0 means poor oral hygiene.

### Standardization of examiner

Prior to the commencement of the study, one of the authors (T.O) underwent a series of calibration exercises. Calibration for the diagnosis of MIH was done using coloured picture chartsof MIH affected teeth, with varying degree of severity of the lesion. This was followed by the use of live patients with MIH. The lesions were graded as mild, moderate and severe, according to the criteria earlier stated. The intra examiner kappa variability score was 0.90.

### Data analysis

The data generated from this study was subjected to suitable statistical analysis conducted with the use of STATA, version 12.0. Descriptive analysis was used to describe the demographic variables (age and sex). The difference in the frequency of observed pathologies in children with and without MIH was determined using Chi-Square test. Statistical significance was established at p values equal to or less than 0.05.

### Ethical consideration

Ethics approval for the study was obtained from the Obafemi Awolowo University Teaching Hospitals Complex, Ile-Ife, Health Research Ethics Committee (ERC/2011/06/03). Approval was also obtained from the Ministry of Education and the Heads of all the schools that participated in the study. Only children whose legal guardian consented to their participation and those who gave assent to study participation were eligible to participate in the study. Each child was examined in an empty room to ensure privacy. All children with oral lesions, with or without MIH, were referred to the paediatric dental unit of Obafemi Awolowo University Teaching Hospitals’ Complex, Ile-Ife, for management. All treatments were offered free.

## Results

Parental consent and assent was received from 2,165 children but only 2,107 (97.3%) met the inclusion criteria. These include 1,125(53.4%) females and 982(46.6%) males. The mean age of study participants was 12.57 ± 2.39 years.

Two hundred and sixty seven (12.7%) children had MIH. Table 1 shows the profile of the study participants. There was no significant difference in the proportion of children with and without MIH by age (p = 0.23) and sex (p = 0.14). Of the 267 children with MIH, 179 (66.9%) had mild lesion, 52 (19.6%) had moderate lesion and 36 (13.5%) had severe lesion.

**Table 1:** **Profile of the study participants**

**Variables**	**MIH absent (%) n = 1,840**	**MIH present (%) n = 267**	**Total N = 2,107**	**p value**
*Age*				
8-10	386 (21.0%)	83 (31.1%)	469 (22.2%)	P = 0.23
11-13	668 (36.3%)	98 (36.7%)	766 (36.4%)	
14-16	786 (42.7%)	86 (32.2%)	872 (41.4%)	
*Sex*				
Male	842 (45.8%)	140 (52.4%)	982 (46.6%)	P = 0.14
Female	998 (54.2%)	127 (47.6%)	1,125 (53.4%)	

Table 2 shows the distribution of the children with and without MIH who expressed concerns with their aesthetics, reported dentine hypersensitivity, had traumatic dental injury and caries and their oral hygiene status. Only children with MIH expressed aesthetic related concerns (19.1%) and had dentine hypersensitivity (15.0%).

**Table 2:** **Associated co-morbidity**

	**MIH absent (%) n = 1,840**	**MIH present (%) n = 267**	**Total (%) N = 2,107**	**p value**
*Oral hygiene*				
Good	879(47.8%)	67(25.1%)	946(44.9%)	<0.001
Fair	675(36.7%)	127(47.6%)	802(38.1%)	
Poor	286(15.5%)	73(27.3%)	359(17.0%)	
*Carious teeth*				<0.001
Caries	109 (5.9%)	68 (25.5%)	177 (8.4%)	
No caries	1,731(94.1%)	199(74.5%)	1,930(91.6%)	
*Sensitive teeth*				
Present	-	40(15.0%)	40(1.9%)	
Absent	1,840(100.0%)	227(85.0%)	2,067(98.1%)	
*Aesthetic concern*				
Yes	-	51(19.1%)	51(2.4%)	
No	1,840(100%)	216(80.9%)	2,056(97.6%)	
*Traumatic dental injury*				
Present	141(7.7%)	26(9.7%)	167(7.9%)	
Absent	1,699(92.3%)	241(90.3%)	1,940(92.1%)	0.241
*Total*	1,840(87.3%)	267(12.7%)	2,107(100.0%)	

Approximately 45% of the pupils had good oral hygiene, about 38% had fair oral hygiene and 17% had poor oral hygiene. More children with MIH had fair and poor oral hygiene when compared with children without MIH while more children without MIH had good oral hygiene. The difference in oral hygiene status of children with and without MIH was statistically significant (p < 0.001).

One hundred and seventy seven (8.4%) children had caries in the permanent dentition. The proportion of children with MIH who had caries, was significantly more than those without MIH and had caries (25.5% vs 5.9%; p < 0.001). The DMFT of children with MIH was 0.5 while the DMFT of children without MIH was 0.1. There was a significance difference in the DMFT of children with and without MIH (p < 0.001).

One hundred and sixty seven (7.9%) children had fracture of the anterior teeth. The proportion of children with MIH who had fracture of the anterior teeth was not significantly more than the children without MIH who had fracture of the anterior teeth (9.7% vs 7.7%; p = 0.24).

## Discussion

This study shows that children with MIH had significantly more oral pathologies when compared with children without MIH: children with MIH reported experiencing dentine sensitivity, had concerns with the aesthetic appearance of their teeth, had more carious lesions and were more likely to have poor oral hygiene status.

This study makes a unique contribution to the growing literature on MIH. Studies such as ours are important because of evidence of regional and racial disparity in the occurrence of dental lesions. Currently, there is no data on MIH from Nigeria: this study provides the first data on MIH in the country. However, the study has three limitations. First, this was a school based survey. This implies that the data generated cannot be generalized to all the children in the study population since a significant number of children in the community do are not in school [15]. The inclusion of children in public and private schools helped to increase the probability of including children from all the socioeconomic status in the study sample. This is important as children in public schools are likely to have low socioeconomic status while those in private schools are likely to have high socioeconomic status [16]. Second, the study population included children between the ages of 11 and 16 years. This age range is larger than the appropriate age for determining the prevalence of MIH. The appropriate age range for determining the prevalence of MIH is eight to ten years [5]. The proportion of children who had MIH in this study can therefore not be representative of the prevalence of MIH in the study population. Third, the diagnosis of MIH and caries and MIH was made using natural light. This may have resulted in the examiner missing some cases of caries and some cases of MIH. Also, the use of the World Health Organisation criteria for the diagnosis of caries also implies that less number of carious lesions could have been detected.

Despite these limitations, the study provides useful information that is important for the clinical management of patients with MIH. The higher prevalence of children with MIH who had poor oral hygiene when compared with children without MIH is an important finding. The poorer oral hygiene may have resulted from increased plaque retention due to the rough surface of the enamel, from poor tooth brushing due to the hypersensitivity and or poor tooth brushing due to the pain associated with the presence of caries. Unfortunately, the poor oral hygiene status may be a mediating risk factor for the higher prevalence of caries in children with MIH. Education about oral toileting, including the use of fluoridated toothpaste twice daily, may be very beneficial for children with MIH: It may serve as a protective factor for caries and poor oral hygiene.

The high incidence of dentine hypersensitivity associated with MIH in this study had been reported in prior studies [6,7]. Dentine sensitivity results from the porosity of the enamel and disorganised enamel rods structure found in MIH [7], from the post-eruptive crown breakdown sequeale to MIH, and from dentine caries. The plausibility that the dentine sensitivity was a result of caries is low, since none of the children without MIH complained of dentine sensitivity.

This study, like other studies [17,18], shows that children with MIH have higher risk for caries. This study fails to report the relationship between post-eruptive breakdown and caries, a relationship that would have shown whether post eruptive breakdown is responsible for the reported high caries experienced in children with MIH, when compared with children without MIH.

The high prevalence of co-morbidities associated with MIH makes it imperative that efforts should be made to promote early diagnosis and management of MIH. While the authors could not find any study on the quality of life and MIH, there are a few studies that have shown that the quality of life of children is negatively affected by caries [19-21], dentine sensitivity [22] and poor aesthetics [23]-three morbidities significantly associated with MIH.

## Conclusion

This study demonstrates that MIH is associated with oral health morbidities that affect the quality of life. Prompt diagnosis and management of MIH, to prevent post eruptive breakdown, can help reduce the co-morbidities identified in this study.
